# Some Multidimensional Unintended Consequences of Telehealth Utilization: A Multi-Project Evaluation Synthesis

**DOI:** 10.15171/ijhpm.2019.12

**Published:** 2019-03-10

**Authors:** Hassane Alami, Marie-Pierre Gagnon, Jean-Paul Fortin

**Affiliations:** ^1^ Institute of Health and Social Services in Primary Care, Research Center on Healthcare and Services in Primary Care, Laval University, Quebec City, QC, Canada.; ^2^ Research Center of Quebec City University Hospital Center, St-François d’Assise Hospital, Quebec City, QC, Canada.; ^3^ Faculty of Nursing Science, Laval University, Quebec City, QC, Canada.; ^4^ Department of Social and Preventive Medicine, Faculty of Medicine, Laval University, Quebec City, QC, Canada.

**Keywords:** Telehealth, Unintended Consequences, Implementation, Evaluation, Healthcare Services

## Abstract

**Background:** Telehealth initiatives have bloomed around the globe, but their integration and diffusion remain challenging because of the complex issues they raise. Available evidence around telehealth usually deals with its expected effects and benefits, but its unintended consequences (UCs) and influencing factors are little documented. This study aims to explore, describe and analyze multidimensional UCs that have been associated with the use of telehealth.

**Methods:** We performed a secondary analysis of the evaluations of 10 telehealth projects conducted over a 22-year period in the province of Quebec (Canada). All material was subjected to a qualitative thematic-pragmatic content analysis with triangulation of methodologies and data sources. We used the conceptual model of the UCs of health information technologies proposed by Bloomrosen et al to structure our analysis.

**Results:** Four major findings emerged from our analysis. First, telehealth utilization requires many adjustments, changes and negotiations often underestimated in the planning and initial phases of the projects. Second, telehealth may result in the emergence of new services corridors that disturb existing ones and involve several adjustments for organizations, such as additional investments and resources, but also the risk of fragmentation of services and the need to balance between standardization of practices and local innovation. Third, telehealth may accentuate power relations between stakeholders. Fourth, it may lead to significant changes in the responsibilities of each actor in the supply chain of services. Finally, current legislative and regulatory frameworks appear ill-adapted to many of the new realities brought by telehealth.

**Conclusion:** This study provides a first attempt for an overview of the UCs associated with the use of telehealth. Future research-evaluation studies should be more sensitive to the multidimensional and interdependent factors that influence telehealth implementation and utilization as well as its impacts, intended or unintended, at all levels. Thus, a consideration of potential UCs should inform telehealth projects, from their planning until their scaling-up.

## Background

Telehealth, defined as “*health care and services, as well as social, preventive and curative services, delivered remotely by means of a telecommunication, including audiovisual exchanges for information, education and research, and treatment of clinical and administrative data purposes,*”^1^ has become an important lever in reform strategies to improve access, continuity and quality of healthcare and services for people, especially those living in rural and remote areas or those living with chronic diseases. For instance, it is seen as an important avenue to address territorial disparities by eliminating the geographical barriers of access to services, while contributing to the emergence of other ways of conceiving the clinical practices and the offer of services.^2-5^ Telehealth could also facilitate better data integration and population health management, reduce wait times for access to adequate services, and reduce hospital stay, emergency room visits, and clinical errors.^4^ In addition, it could also contribute to developing better interprofessional collaboration, particularly in the management of chronic diseases, and improving team work by increasing the collective commitment and continuous learning of health professionals.^6^ Telehealth could also help reduce professional and geographic isolation and develop better clinical skills.^7^

Nonetheless, telehealth is a complex enterprise that raises multiple questions and challenges. Indeed, it involves a set of multilevel sociopolitical, economical, organizational, professional, cultural, human, legal, technological, and strategic factors.^5,8-10^ However, not all of these factors are usually taken into account in the planning, implementation, deployment and evaluation of telehealth projects, although they bring along numerous changes and transformations at the micro, meso and macro levels.^11-13^ Over the last years, rapid technological advances have led to the implementation of a variety of telehealth solutions. These are often perceived as a panacea, without extensive hindsight and anticipation regarding their potential impacts and consequences, including unintended and negative ones.^12,14,15^

The concept of “unintended consequences” (UCs) refers to a set of outcomes that result from the introduction of an innovation within an organization or a social system.^16^ These consequences can be positive (eg, improving diagnostics), negative (eg, increasing stress), or neutral (eg, maintaining efficacy). In any case, they are not planned by the stakeholders concerned by the innovation in a given context. These consequences, positive (happy surprises) or negative (lessons sources), characterize the side effects of socio-technical systems.^17^ These effects can be directly attributable to technology, or indirectly linked via a chain of events that causes such effects (indirect causal chain).^11^

In the health sector, UCs related to information and communication technologies (ICTs) have mostly been considered through errors related to the use of the technology and resulting clinical harm to patients.^14,15^ The term “*e-Iatrogenesis,*” which refers to the damage caused to the patient, at least part of which is related to the use of health-ICTs, is increasingly used.^11,18,19^ Most studies that have documented such unintended negative consequences of ICTs involved decision support systems, computerized provider order entry systems, and medication administration systems.^11,20-24^

For instance, Campbell et al have identified nine types of UCs that could be related to computerized order entry and decision support systems.^25^ These UCs concern increased workload, changes in workflows, new demands related to the technology, persistence of paper, changes in communications, negative emotions, new errors, modification in power structure, and overdependence on technology. For their part, Bloomrosen et al indicate that it is very difficult to develop a unique taxonomy of the UCs of innovations in general.^11^ This could be explained in part by the fact that theoretical and conceptual developments on the issue of UCs of innovations are almost non-existent.^26^

With respect to telehealth projects, our search of the scientific literature found very few studies that have explicitly considered their UCs.^12,27^ This is a somewhat surprising finding given that telehealth could be associated with important changes in the health system, but also with a lot of uncertainty regarding its impacts on healthcare and health outcomes. Previous work clearly shows the importance of adopting a multidimensional and holistic perspective upon the UCs of telehealth in order to better account for the complexity of its integration into healthcare and services.^12,19^ Such a perspective could also help to innovate and to prevent, or at least to attenuate, some harmful consequences of telehealth utilization.^20^

The aim of this paper is to explore, describe and analyze a set of multidimensional UCs that have been associated with the use of telehealth by analyzing data from the evaluation of 10 telehealth projects conducted in the province of Quebec (Canada) over more than 2 decades (1993-2015). Each project covered a distinct telehealth solution or population and involved various stakeholders, from primary to specialized care. However, all these projects share a similar comprehensive evaluation approach that makes it possible to account for their complexity. This secondary analysis focuses on exploring their UCs at various dimensions, which could provide important and useful knowledge to inform future telehealth developments and evaluations.

## Methods

### Study Design and Selection of Projects

We conducted a secondary analysis of the evaluations of 10 telehealth projects conducted over a 22-year period (1993-2015) in the province of Quebec. Secondary data analysis allows the interrogation, re-exploration and re-use of data collected in the past and whose results extend, and often differ, from those of original analyses that may have different objectives.^28^ A wide variety of data sources could be subject to secondary analyses, including, but not limited to interviews, surveys, and databases.^28,29^ One of the practical interests of secondary analysis is pragmatic in that it exploits data that are difficult to collect in a primary way.^29,30^ It could be an exploratory step prior to further studies on a given issue.^29,30^ Secondary analysis makes it possible to reconstruct logics and make discoveries, sometimes by surprise, thanks to the possibility of going beyond the limits of knowledge available at the time of primary data collection.^29^

The projects were selected because they are major telehealth projects in which the authors were involved, especially in the evaluation, making it possible to access complete and rich data about each project. They cover a vast array of clinical specialties and technologies: psychiatry, pathology, oncology, pediatrics, radiology, cardiology, ophthalmology, diabetology, pre-hospital emergency care, primary care, and general medicine (Table 1). All these projects were conducted in the same institutional environment, the Quebec health system, which increases the consistency and complementarity of the results because the projects share a set of issues and constraints even though reforms of the health system happened during that period. This makes it possible to cover a multitude of dimensions from different angles while addressing the issue of comparing evaluation results from different environments and countries.^31^

**Table 1:** Description of the Telehealth Projects Included in the Study

**Projects**	**Overview**
The Eastern Quebec Telepathology Network (2004-2015) (A)	Network of different specialists (eg, pathologists and surgeons) and organizations to ensure access and continuity of pathology services throughout eastern Quebec, thereby avoiding the transfer of patients and interruption of services in rural and remote areas. (Still functional).
"My digital primary health care" (E-personal health record E-PHR) (2012-2015) (B)	By optimizing communication between patients with chronic diseases and interdisciplinary care teams, the project aimed to improve access, quality, and continuity of primary healthcare and services in order to make patients more active in the management of their health and disease (No longer active).
Teleophthalmology (diabetic retinopathy) (2010-2013) (C)	Designed to provide diabetic retinopathy screening services remotely for Aboriginal and First Nations communities in Quebec, the project also aimed to strengthen service corridors in order to ensure the follow-up and management of diabetic patients (Still functional).
Telemedicine in pre-hospital emergency services (2006-2011) (D)	This project linked nurses and nurse practitioners to emergency physicians in order to support, in real time (using ICTs), ambulance technicians/paramedics. The aim was to optimize the ambulance transport time for patients with an unstable state of health (Still functional).
"CLSC of the Future" (Telehomecare and telemonitoring) (2000-2004) (E)	A network that facilitated information-sharing and decision-making between clinicians and patients, supporting services integration and coordination of in-home care (No longer active).
The Quebec Oncology Computerized Network (1999-2002) (F)	This electronic health record system for clinicians who provide care for women with breast cancer was designed to support the delivery of integrated oncology care by improving the flow of clinical information among caregivers and between the caregivers and their patients (No longer active).
"Telemedicine for regions" (Teleconsultation) (1997-2000) (G)	By improving communication between teams of professionals, the project aimed to improve access to specialized health services for remote populations (teleconsultation) and access to training for the professionals practicing in these regions (teletraining) (Still functional with minimal activity).
Telepsychiatry (H) (1998-1999)	Designed to provide outpatient psychiatric services for patients referred by a family doctor who took charge of the patient’s follow-up, this project allowed remote access to a psychiatrist, which avoided travel for both patients and psychiatrist (No longer active).
Pediatric telecardiology and teleradiology (1995-1998) (I)	Designed to provide specialized distance pediatric cardiology and radiology services for rural and remote areas that didn’t have radiologists or pediatric cardiologists on site. Therefore, it reduced wait times for patients or avoided their transfers and travel over long distances (Still functional with minimal activity).
The Rimouski Microprocessor Health Card (1993-1995) (J)	Designed to ensure that patients and healthcare professionals used a microprocessor card that provided quick access to the patient’s clinical and administrative information (No longer active).

Abbreviations: E-PHR, E-personal health record; ICTs, information and communication technologies; CLSC, Centre local de services communautaires.

### Conceptual Model

We applied the conceptual model of the UCs of health information technologies (health-ITs) proposed by Bloomrosen et al.^11^ This model was proposed to better understand the complexity of the different forms that the UCs of health-ITs may take. It covers four domains: (1) *Technology:* the transition from a physical (eg, paper) to a virtual environment is accompanied by significant changes. We can see new types of errors emerging caused by, among other factors, rigid processes imposed by technology, ergonomics, and design or new modes of virtual communication that differ from physical contact (face-to-face); (2) *Human factors and cognition:* the question of human-technology interaction can have important cognitive and human implications concerning knowledge, habits, behaviors, memory, mental and cognitive elements, psychomotor factors, and individual psychosocial and cultural characteristics. Technology is a set of artifacts which must take into account the particularities and characteristics of users; (3) *Organization:* healthcare organizations are complex social systems, with heterogeneous and varied individual and group cultures, dynamics, interests, and behavior. Health-ITs, as a sociotechnical object, could trigger a redistribution of certain equilibriums, workflows, and powers, thus creating professional and organizational jurisdiction conflicts, etc; (4)* Fiscal/policy and regulatory:* the health sector is highly regulated. The evolution of standards and certification requirements and obligations (eg, quality, safety, and privacy), financial and economic frameworks, and various initiatives and policies related to health-ITs (or ICTs in general) can engender a vast array of UCs.

### Data Collection and Analysis

We conducted an extensive literature search (paper and electronic documents) in the archives of the Senior Evaluation Officer (JPF), via internet and in other sources related to different projects (eg, organizational documents, government documents, publications). This approach helped find documents related to the projects: evaluation reports, published articles, policy briefs, papers and presentations, project planning, project operation manuals, protocols and guidelines, minutes of meetings, activity tracking and monitoring reports, newsletters, observation notes, field notes, interview transcripts, and documents from government and funding agencies. All this documentation enabled an in-depth re-analysis of each project with a specific focus on their UCs.

All material was subjected to a qualitative thematic-pragmatic content analysis.^32-34^ Thematic analysis consists of identifying, classifying and combining data in order to distinguish themes and to relate or integrate them with others.^32-34^ The pragmatic dimension refers to the interpretative and emerging aspect of the data. Indeed, during the data analysis process, we used the comments of co-researchers or project-related people that could be used to complement the analyses. The reference to the literature could also be used to obtain information supporting the identification of UCs.^32-34^ In this regard, we used a deductive-inductive approach, based on the UCs model^11^ and new themes emerging from the data.^35^ This approach allows a hybrid codification where the dimensions that make up our model and the new emergent themes form the themes retained.^31,34^ All the analyzes were performed, discussed and reviewed by HA, MPG, and JPF, which allowed us to reformulate, merge or add themes as needed.^32,35^ Following the first analysis step, an additional coding cycle was conducted to validate the final themes in a consensual manner.^36^ This approach allowed discussing, qualifying, contextualizing, completing and validating the results during deliberations of the members of the team (HA, MPG, JPF). In the end, the components of the UCs framework were kept in addition to the clinical and professional dimension that the researchers found important to distinguish. We applied the principles of data triangulation at 2 levels: (1) methodological triangulation, which involves the use of multiple data collection techniques (eg, interviews transcripts, projects documents, observation notes, field notes, documents from government and funding agencies); and (2) triangulation of data sources, which results in a search for information from different actors and stakeholders during the evaluation of each of the projects studied.^37-39^ This strategy made it possible to formulate, complete, and revise our findings, and to compare and contrast multiple sources by returning regularly to primary data sources in order to verify divergences/convergences and detect differences or gaps (eg, check for discrepancies or contradictions that may exist between organizational or government documents and what was reported in the verbatim reports of the stakeholders interviewed).^32,40^

## Results

In light of the inductive-deductive analysis using the UCs model and new themes that emerged from the data, we present the results according to the following domain taxonomy: (1) technology (technology and technology providers); (2) human and cognitive; (3) clinical and professional; (4) organizational; (5) legal, regulatory, political and social; and (6) economic and financial. The letters in parentheses refer to the project(s) in which the UCs were documented (see Table 1). Table 2 presents a summary of all the domains covered.

For the following, the results and findings are presented narratively.

**Table 2:** Synthesis of Some UCs That Emerged From Telehealth Projects

**Domains**	**Observed UCs**	**Potential UCs**
Technological	• Huge storage and archiving needs: images, video, etc • New forms of errors: mixture, truncation, or loss of information • Incompatibility of technological standards between jurisdictions or countries: safety, quality, etc • Several software components in the same system: quality control and security • Saturation or insufficient bandwidth problem in rural and isolated areas • Technological dysfunction: negative impact on the image and reputation of organizations and clinicians	• Attempted monopolization by some technology providers: risk of dependence of organizations, clinicians, or patients upon these suppliers and difficulty with respect to evolving or changing technology • Rapid change and evolution of technology: less time for organizations, clinicians, and patients to become familiar with and adapt to such shifts
Human and cognitive	• Growing dependence of clinicians and patients on technology: alert fatigue, anxiety, stress, etc • Decontextualized information: increased anxiety of the patient if no "e-literacy" and "clinical-literacy" • Feeling of isolation on the part of health professionals: loss of physical contact with patients and colleagues (eg, corridor discussions and informal relationships), loss of a feeling of belonging to the organization • Technological rigidity less adapted to the reality of clinical practice ("technology-driven"): frustration, stress, development of circumvention strategies, and risks of errors • Medicalization and intrusion into people’s living space and privacy: technology as burden • Cognitive overload: handling of large amounts of data by clinicians and patients	• Depersonalization of the clinician-patient relationship: reduction of contact time and increased detachment • Risk of diversion of the technology from its clinical function to a control tool of patients or professionals
Clinical and professional	• High resolution images and large amount of data: overinterpretation and overdiagnosis • Increased data flow and diagnostic capacity that can affect forensic liability • Non-transfer of patients: increased complexity of clinical cases in small hospitals that do not necessarily have expertise to take care of such cases • Non-integrated data: obligations to address fragmented data from different systems, duplicate tasks, increase in clinicians’ workload, etc • Professional jurisdictions and professional equilibriums: reserved acts, new expertise, professional collective agreement, etc • Emergence of new unplanned clinical uses of technology: expansion of the range of services offered by the organization	• Clinical interoperability (between organizations or jurisdictions) and need of protocol standardization, standards of practice and diagnostic methods: risk of hampering innovation and local creativity • Easier access to specialists and experts via telehealth: risk of loss of expertise and culture specific to practice in rural and remote areas
Organizational	• Restructuring of hierarchical relationships within organizations: clinician-clinician, clinician-other professionals, clinician-organization, etc • Standardization of human resources management: staffing (allocation) and unions (associations) • Strategic positioning of organizations: competition and tensions between organizations with respect to concentrating services and increasing revenues • Impact on the distribution of medical staff within the jurisdiction: tendency to concentrate medical expertise in large centres, loss of human resources and difficulty in recruiting and retaining these resources in small rural hospitals • Non-transfer of patients: an additional need for human resources (clinical and administrative) for small hospitals to provide care and services • Changes in the organization of services and professional work: prioritization of internal service requests vs. external requests	• Direct accessibility to specialized and subspecialized services: bypassing and disorganization of traditional service corridors, inflation of requests for expertise, misuse of services, increase in wait times • Modification of pre-existing professional and organizational collaboration networks if telehealth is developed without taking them into account
Legal, regulatory, political, and socia***l***	• Dilution of responsibilities due to the multiplicity of stakeholders: clinicians, technology providers, organizations, etc • Legal responsibility of clinicians to use data captured by the patient to make clinical decisions • Delegation of medical activities: need for agreements between professional associations and orders, provincial and federal ministries • Emergence of new modes of practice (smartphone, work or monitoring from home): insurance issues, quality control, labour standards, etc • Central role of insurance agencies (professional risk coverage): recommendations and requirements that are difficult to apply by professionals and organizations • Law on the exchange of personal data and information: obstacle to implementing a "public-private" telehealth network or archiving and sharing of patient data outside Quebec (eg, cloud computing) • Conflicts and inconsistencies of missions between levels of governance: provincial vs. federal vs. communities • Commercial use of patient data: consumer data or health data? Property of the technology provider or that of the patient or organization?	• Practice permit for foreign clinicians: risk of prosecution for illegal practice of medicine • Intellectual property of new uses of technology made by clinicians or patients • Package of technologies and software components from different manufacturers, multiplication of subcontracts: liability in case of damage, compliance with regulatory, quality, and safety standards • Outsourcing and "subcontracting" of certain technical assistance services in other countries: unauthorized external third parties may have access to patient data
Economic and financial	• Cost-sharing (eg, maintenance, storage, operating costs, human resources) and redistribution of benefits between organizations and even jurisdictions • Organizational performance criteria not adapted to telehealth: accounting for activity vs. costs of physical care of the non-transferred patient • Non-transfer of patients: increase of expenses and operation costs for its management in the organization • Strategic positioning and competition between organizations: accounting for the telehealth activity without having to assume the costs of physical care of the patient and competition for "market shares" • Additional costs for some organizations: upgrading technology and infrastructure to align with other participating organizations • Harmonization of salaries or remuneration of clinicians from different organizations or jurisdictions • Displacement of professional jurisdictions: enhancement of remuneration • Opportunism of some technology providers (fees and additional purchases): increased expenses for patients and organizations • Additional expenditure for the health system: ambulance transport companies that increase rates to compensate for the shortfall, etc.	• Circumvention of service corridors: demand inflation for specialized services and increased spending on the healthcare system • Outsourcing of medical activity: problem of the health system financial flows destination • Increased workload of family caregivers (eg, telehomecare): financial compensation by insurance companies for caregiver time

Abbreviation: UCs, unintended consequences.

### Technology (Technology and Technology Provider)

#### New Technological Needs

Innovative uses of telehealth have raised the need for larger spaces and capacities to ensure the storage of diagnostic quality images (eg, image x20 = 2Gb) and videos. New videoconferencing cases were considered sufficiently sensitive (or a source of potential litigation, especially in case of medical error, thus to define the responsibility of the surgeon, technologist or pathologist) to be recorded and archived, which requires huge space for storing videos (A).

#### Overconfidence in Technology

Problems of overestimated performance and maturity, in addition to the cohabitation of several non-integrated applications and systems (eg, physician’s record, patient’s record, pharmacist’s record, systems of other organizations), were sources of surprises. For example, the non-compatibility of 2 language versions (French-English) of the same application has been observed for an interjurisdictional telehealth network (E). Thus, the exchange and sharing of data was confronted with certain difficulties.

In some situations, clinical data (eg, laboratory results) of some patients were sent by error to others, or patients received clinical results that had not been previously checked, validated, and authorized by the clinician (B, F). These incidents caused the reluctance of clinicians and patients to use the technology afterwards.

#### Rigidity of the Technology

In some cases, the technology was poorly adapted to the complexity of medical practice, in particular with regard to the uniqueness of each clinical case and the different modes of practice according to the contexts. Some technology providers tended to prioritize major scenarios that left little flexibility for clinicians, who were forced to align with rigid processes imposed by technology, and that were not adapted to their practice (“*technology-driven*”). For example, to ensure workflow continuity, clinicians were obliged to update their systems on a regular basis. In addition, systems shut down automatically after a period of inactivity or had to be interrupted during update or maintenance periods (B, E, F, G).

#### Decontextualized and “Multi-origin” Technology

Some telehealth devices contained software components from more than a dozen different technology providers (eg, pharmacy, laboratory, clinic, and billing), which complicated matters when it came to upgrading the technology while ensuring respect for quality and safety standards. The situation was even more complex when several organizations were involved in a telehealth network where each had its own infrastructure and technological history, with systems that were not necessarily functional or interoperable elsewhere (A, B, F, H, J). In addition, some of the technologies used were developed without taking completely into account the reality of users (eg, the constraints of operating on limited internet bandwidths in rural and isolated areas) (B, E, G, I).

#### Dependence on the Technology Provider

On many occasions, the technology did not offer the possibility of integrating other applications or new technologies, especially from other vendors (A, B, E, F, J). The problem of interoperability could be explained by the desire of some technology providers to have a monopoly and to make organizations dependent on their own products, even if other competing technologies were available. This power relation has been observed in some attempts to monopolize the computerization of services by eliminating any competition or the use of systems developed *ad hoc* in some organizations (B, F, E, I, J). This situation is likely to cause a dependence on a single technology provider in a position to impose its own conditions.

### Human and Cognitive

#### Dematerialization and Depersonalization of Relationships and Interactions

Telehealth entails significant changes in modes of practice and communication along with new types of relationships between professionals and technology and between patients and technology. Telehealth also causes the loss of material (eg, 3D samples vs. 2D photo) and physical (eg, physical examination of the patient) dimensions, thus necessitating an adaptation of the cognitive processes of information processing and communication (virtual communication) for clinicians and patients alike. On this point, the question of the depersonalization of the clinician-patient or clinician-clinician relationship arose in several projects (B, D, I, F, H).

For patients, the technology was sometimes seen as reducing the time of contact with the clinician (clinical time) (B, E, J). In addition, the consultation could become more technology-centred and less patient-oriented, leading to a sense of depersonalization of the consultation.

For some clinicians who provide on-call distance or cover the services from their homes, the loss of physical contact with patients or colleagues (eg, corridor conversations, team meetings, or informal exchanges) has led to a feeling of isolation. The same situation was also noted among other clinicians, affiliated with different organizations, who participated in telehealth networks based on virtual teams; they could hardly develop a sense of belonging to this new “dematerialized” organization-network (A, E).

#### Alert/Alarm Fatigue, Cognitive Overload, Burden, Stress and Anxiety

Dependence on technology, for both clinicians and patients, was reported regularly (B, D, E, F, H, I, J). In some cases, dysfunction of the technology increased stress, anxiety, and discouragement (eg, the obligation to repeat the same information to ensure that it was well understood). For example, in telehomecare, clinicians had to travel in response to “false alarms” coming from people’s homes. Some of these alerts were caused by technological malfunctions or bugs (eg, problem of parameterization) (B, E, F). The recurrence of these false alarms could lead to the development of a phenomenon of tolerance in clinicians, also called alert fatigue, which could lead to a voluntary disabling of these warning systems and could be dramatic in case of no reaction to a true alert.

For their part, some patients became dependent on technology in their living space. Cases of stress and anxiety related to self-measurement and self-monitoring have been reported (eg, patients who had to monitor the value of their blood sugar or blood pressure in the system several times a day) (B, E). This dependence often made technology more a burden than a solution and led to an over-demand on the clinical team (eg, telephone calls, messaging, request for appointment) whenever a patient was confronted with unusual information or measures.

In addition, the continuous flow of data enabled by the technology involved handling increasing amounts of information that could not be processed and analyzed in real-time. This “cognitive overload” could result in errors or prejudices (B, E, F, J).

The issue of medicalization of living space with technology intruding upon individuals’ privacy has been reported (B, E). The concerns regarding the increased burden on family caregivers, who have taken on some of the clinicians’ tasks, have emerged (B).

#### Feeling of Control Among Patients and Professionals

The potential risk of diversion of the technology from its clinical function to a control tool, whether for patients or professionals, has been raised (C, D, F, J). For example, concerns were expressed about the possibility of provincial and federal governments accessing population data for evaluation purposes, creating a fear that these data would be used to implement other policies or programs that may adversely affect the community (eg, cultural or ethnic communities).

### Clinical and Professional

#### Increase in Workload and Modification of Workflows

Problems of interoperability between systems, in addition to the coexistence of several non-integrated applications (eg, physician’s record, patient’s record, pharmacist’s record), also led to a duplication of tasks for clinicians who had to seek information dispatched in different systems. The same was true for the data capture of several non-interoperable softwares, resulting in duplicated, altered, or incomplete data (A, D, E, F, G, J).

Telehealth also led to changes in work processes and the emergence of new medico-administrative workflows (eg, aligning schedules and coordinating appointments) (B, C, D, E, F, H, I, J).

#### Overdiagnosis and Overinterpretation

The technology offers a quality of image resolution that could push clinicians to overinterpret and overdiagnose (eg, digital image vs. physical glass under microscope) (A, I). Clinicians emphasized that physiological parameters and clinical data were sometimes difficult to interpret when decontextualized (eg, lack of information on physical activity, diet, or sleep, or no actual observance of the drug). However, in situations where these data have been mined intelligently, it has been possible to detect other unknown (or even unsuspected) problems in some patients (eg, cardiac problems, asthma, and hypertension) (B, E, I).

#### Dilemma Between Standardization of Practices and Local Practice

The proliferation of local models and protocols has raised the fear of fragmentation of services in the province (A, D, G, I). However, the idea of too much standardizing protocols, even when necessary, has also been seen as a risk in terms of slowing down or constraining the potential for innovation, or adaptation, and the introduction of new knowledge and discoveries among local clinical teams. Indeed, in some cases, the creativity and inventiveness of local teams may have allowed other clinical uses (not initially planned) for technologies that were originally designed for a particular service or application (eg, telepathology to do teleautopsy; telecardiology to do teleorthopedics), making it possible to expand the coverage of services to supplement patient care in some small rural hospitals (A, E, G, I).

On another scale, direct and easy access of rural physicians to specialists in urban centres was also be seen as having a negative impact on the potential for clinical innovation and creativity in small rural hospitals. Indeed, there is concern that dependence on large centres may result in the loss of a specific expertise and culture in rural areas where, in the absence of specialists, teams often develop local solutions to problems (D, E, G, H, I). The risk of unavailability of local expertise in case of technical problems could cause an interruption of services in these environments.

Problems of clinical interoperability have also been observed in inter-regional or inter-jurisdictional telehealth networks (eg, different clinical protocols, different diagnostic methods) (A, D). The standardization of protocols and diagnostic methods in these networks has been confronted with the necessity for clinicians and organizations to apply specific clinical protocols to deliver services via telehealth while providing traditional services for their local clients.

#### Increase in the Complexity of the Clinical Cases in Small Hospitals

On some occasions, the non-transfer of patients from rural hospitals as a consequence of telehealth increased the complexity of cases hospitalized locally. However, these small hospitals did not always have the clinical and paraclinical expertise necessary to ensure complete management and support of these patients at a local level (G, I).

## Organizational

### Changing Dynamics and Hierarchical Relationships

Telehealth has led to major changes in interprofessional, interorganizational, and professional-organization dynamics, balances, and relationships. For instance, in telepathology, replacing the microscope by digital technologies put the organization in a position of strength, because it had the financial means to buy and update the technology (A). Telehealth could thus result in the renegotiation of the agency relationship and “information asymmetry” that linked the organization with clinicians, here the pathologists. Some clinicians had rightly maintained that the organization, by controlling the technology, would interfere more in their activities, thus questioning the principle of professional autonomy.

Telehealth also resulted in changes and shifts in the contours of professional jurisdictions (eg, reserved activities). Examples include a nurse performing echography, a technologist performing an autopsy under the supervision of a pathologist, a general practitioner or internist performing an act reserved for cardiologists, and an ambulance technician who dispensed opiates in place of the emergency physician (A, B, C, D, F, G, I, J).

In such situations, the need to recognize and value the new expertise emerged. This point was a regular source of tension and power relations between the various professional orders (or unions) and between these and organizations. These orders usually wanted to defend the professional jurisdiction and privileges of their members or even negotiate other roles and benefits to compensate those delegated to others. At the same time, there could be a shift of responsibilities associated with the new activities to be carried out, which brought additional training needs to formalize new roles and ensure compliance with practice and quality standards.

Some of the previous issues were also raised in the case of interregional or interjurisdictional telehealth services, particularly in fields of practice that may vary from one jurisdiction to another (A, G, I). For example, in a given jurisdiction, an internist can perform activities as a cardiologist, or a pharmacist can make a medical diagnosis, which is not the case in other jurisdictions. Authorizations for access to certain medico-administrative information and data for some health professionals may also differ according to the organization, geographical area, or jurisdiction in which they practice.

### Strategic Repositioning of Organizations

Unintended organizational dynamics and behaviours were observed, especially in the perspective of a collaborative telehealth network (A, C, D, F, G, H, I). In some cases, hospitals have done strategic repositioning, for instance by concentrating the service in a larger hospital that recruited specialists from other smaller hospitals in order to become the reference provider of the specialized service for the whole health region. These same specialists were able to cover their former hospitals via telehealth afterwards. Indeed, competition between hospitals for the status of “reference centre” of the telehealth network could be explained by the nature of the financial and human resource advantages that organizations can have, but also by the will of certain hospitals to have a distinctive “advertising label” that allows them to have a privileged position. Thus, the fact that telehealth contributed to clinicians leaving small hospitals or closing down laboratories (eg, pathology and medical biology) increased the difficulty of some of these hospitals had in recruiting and retaining staff. This concern with losing local expertise and know-how and with the emergence of large urban centres that concentrate expertise has pushed some rural hospitals to refuse to use telehealth, despite the expressed need, for fear of becoming dependent on these mega-centres (A, G, H, I).

### Organization of Services, Staff Management, and Clinical Performance

In the majority of projects, significant changes in the organization of services and professional work were observed. For organizations providing telehealth services, the increased demand required additional clinical and administrative human resources. This situation has placed some organizations in a dilemma: should they prioritize requests for internal services, even the least urgent ones, or should they respond to requests from outside as they would internally? Indeed, systemic performance criteria required clinicians to respond in priority to requests for internal services from their organizations, even the least urgent ones, before agreeing to cover requests from outside, even for more urgent ones.

In addition, telehealth consultations were recognized to be longer some cases (the whole process of preparation, planning and consultation) (A, C, D, E, G, I). The performance criteria for institutions were not adapted; the productivity of clinicians (paid mainly by activity) and organizations was impacted. In telepathology, the extemporaneous services may take longer than if the pathologist was physically present in the operating room. This has led to increased time of surgery resulting in additional costs for the organization, impacting on its performance and productivity criteria (A).

In the same vein, the need for additional clinical resources in hospitals providing telehealth services has had a direct impact on the distribution of medical staffing plans established by the ministry of health (A, G, H, I).

### Disruption of the Service Corridors and “Inflation” of Demands

In Quebec, primary care services are the gateway to the health system. Thus, specialist consultations must be prescribed by a general practitioner. The direct availability of specialized and subspecialized (secondary and tertiary) services via telehealth has meant that, in some cases, traditional service corridors were not respected or even circumvented (D, G, H, I).

This direct access to specialized services could also lead to demand inflation, thus disorganizing corridors and increasing wait times for those who really need services. Moreover, even for certain cases usually handled locally by interdisciplinary consultation, some physicians have appealed to specialists via telehealth, sometimes to solicit other expertise or second opinion (D, G, I).

### Fragmentation of Services and Disruption of Existing Dynamics

Most telehealth projects have been developed on the basis of specialties or interpersonal links without real comprehensive perspective, interconnections or relationships with other existing services or applications (silos). For example, telepathology was first established to cover breast cancer, whereas the service could be used for other pathologies in gynecology, microbiology, or dermatology (A). This raises the issue of the risk of developing several specialized services in silos.

Conversely, other projects developed have influenced existing professional and organizational functional collaboration networks, which has sometimes weakened some local dynamics that worked well (A, B, E, F, G, H, I).

### Legal, Regulatory, Political, and Social

#### Responsibility and Accountability

All projects highlighted the issue of the “shared secret” as part of collective care of patients via telehealth. Indeed, the multiplication of stakeholders (clinical, administrative, and technological) who may have access to patient data could lead to a dilution of responsibilities, in particular when it comes to engaging professional responsibilities in case of harm to the patient. The legal responsibility of some health professionals has also been questioned regarding certain data and information entered by patients on their electronic records (B, E).

In addition, telehealth often uses a “package” of technologies and software components that can come from different providers. In the event of malfunctions, it is difficult to define the responsibility of technology providers. This situation is complicated by the multiplication of subcontracts (eg, medical data storage or technology maintenance services). The challenge remains to ensure that all these technological and software components comply with regulatory, security, and quality requirements. As some technology providers or subcontractors may be located abroad, the question of the place of storage of medico-administrative data also arose (A, B, C, E, F, I). For instance, Quebec law requires that “physical” data storage and archiving must be done within the province itself. However, some “cloud” service providers are unable to guarantee the actual location of data storage for which they are responsible.^41^

#### Licence to Practice, Practice Modes, and Insurance

On another scale, the issue of licence and authorization to practice arose for physicians from other provinces or countries (A, C, I). The risk of prosecutions for illegal practice of medicine in Quebec for clinicians from other jurisdictions has prompted some Quebec physicians to solicit the expertise of their foreign colleagues in an informal manner. For these clinicians, it was the only way to benefit from such expertise without putting their colleagues at risk of prosecution (A).

In addition, the emergence of new modes of practice (eg, smartphone use, telework) or new uses of technologies has highlighted issues related to insurance coverage (eg, workplace, professional risk coverage), practice quality control and compliance with labour standards (eg, ergonomics) and the actual number of hours worked (A, E, G, I). Indeed, potential security and liability risks arose with these new modes of practice and the use of technological equipment for purposes other than the original one. On this point, the central role of insurance companies and professional orders in making recommendations (eg, quality, practice license, place and duration of image and video storage) was highlighted.^42^

#### Professional Jurisdictions and Reserved Activities

Quebec and Canadian laws and the positions of professional orders (or unions) could be important constraints regarding professional boundaries and the realization of activities reserved for a type of health professional by another (A, B, D, E, F, G, H, I, J). For example, in pre-hospital emergency care (I), ambulance technicians were handling and dispensing opiate products remotely under the supervision of the emergency physician. However, this activity is reserved for physicians by law, and the handling of opiates requires authorization from the federal ministry of health. This new situation resulted in changes in clinical protocols and a transfer of responsibilities between ambulance technicians and emergency physicians. A relatively similar approach was necessary to allow ambulance technicians to file a death report remotely under the supervision of a physician, without being obliged to move the body to a hospital, sometimes over long distances.

#### Technology and Data: Ownership and Use

Misuse of some patient data and information for commercial purposes was reported. For instance, a technology provider used patient information to send advertisements by e-mail, which raised the issue of ambiguity concerning ownership of information: is this information defined as medical data (patient property) or simple consumer data for a commercial service (technology provider property)? In addition, in the context of “public-private” partnership, the question of intellectual property of some new uses and improvements in technology made by clinicians arose: should these new applications be owned by the technology provider or by the clinicians or their organization? (A, B, E, F, J).

#### Technology as a “Political Object”

Telehealth has been source of some tensions between the federal and provincial ministries of health. As health policies in Canada are under provincial jurisdiction, conditional funding of certain telehealth projects by the federal government has sometimes been seen by the Quebec government as an attempt to interfere (A, E, F, G, I). Otherwise, some projects also shed light on the debate on the “public-private partnerships”: whether technological development should be done by the internal teams of the health system (health ministry or hospitals) or by private companies (A, B, J).

#### Outsourcing of Medical or Assistance Activities

The fear of seeing a trend towards the outsourcing of some medical services, in particular radiology and pathology, to foreign countries has also emerged. This fear was reinforced by the revelation in the media of cases of hospitals outsourcing radiology services to other countries via teleradiology in the province of Ontario (Canada).^43^

In addition, situations where technological assistance services to patients and clinicians were provided in languages other than the usual language were identified (B). This situation raised the problem of the use of technological devices for which maintenance and assistance services are provided by subcontractors located in other countries or jurisdictions. The risk that other unauthorized third parties may have access to the medico-administrative data has thus emerged.

### Economic and Financial

#### Cost-Sharing and Savings Redistribution

Cost-sharing and savings redistribution between organizations, and even jurisdictions, was an issue in several projects (B, C, D, E, F, G, I). It was found that performance criteria for organizations were not always well adapted to telehealth. In the current Quebec healthcare system, only the hospital and clinicians that provide the service can include the activity in its accounts, which excludes expenses for patient care that occur at the distant site. Thus, hospitals that request telehealth services bear the costs for the non-transferred patient (A, G, I). This decentralization of care to small hospitals also leads to an increase in the gravity of the cases treated locally, which further increases operating costs (A, G, H, I). However, savings for the healthcare system (eg, reimbursement of ambulance or travel expenses) are not necessarily redistributed to the small hospitals. Furthermore, some large hospitals have shown a willingness to focus expertise and provide services remotely to small hospitals. This status would allow them to benefit from additional financing, material, and human resources without assuming the costs of physical care of patients (A, H, I).

#### Enhanced Remuneration of Professional Activity

The issue of remuneration enhancement for professionals who carry out activities traditionally reserved for others has also been raised. The recognition of this new expertise has sometimes been decisive to ensure the continuity of a telehealth service. Similarly, in inter-jurisdictional telehealth projects (C), clinicians required harmonization of remuneration for inter-provincial consultations because remuneration was higher in one province than the other.

#### Additional Expenses for Patients

In telehomecare and chronic care telemonitoring, patients may incur additional expenses for some technologies (eg, glucometer compatible with the technology used) that are not reimbursed by public health insurance (B). In addition, the need for frequent updating, due to the rapid evolution of technology, has been seen as potentially leading to further costs for patients in the future but also accessibility to healthcare.

#### Additional Expenditures for Organizations

Telehealth has resulted in additional expenditures for some organizations more than others (eg, upgrading technological infrastructure, replacing technologies developed ad hoc, or securing certain technologies). This has created tensions since these organizations felt they had to incur higher expenses compared to others (A, B, E, F, I). Similarly, interoperability problems between organizations (eg, with different technology providers) also meant additional costs that were not equitably distributed between them. Spending on rapid technological change was also a major challenge.

#### Additional Expenditure for the Health System

The direct access to specialized services made possible with telehealth could increase health system expenditures since such services are more costly. Another economic consequence of the decrease in patient transfers and travels associated with telehealth has been the negative impact on the activity of ambulance transport companies. To compensate for this shortfall, these companies have proportionally increased their rates. The loss of ambulance transportation jobs in some small towns was also raised (G, I).

Finally, the risk of outsourcing services via telehealth to other countries or jurisdictions has raised questions about the reimbursement of extraterritorial consultations by the public health insurance system.

## Discussion

This study identified a multitude of UCs resulting from the implementation and utilization of telehealth. The complexity – and the vast diversity – of the UCs that may result from the use of telehealth could be explained by the fact that it is introduced into social systems that have expectations, needs, and sometimes divergent and even contradictory goals, as well as dynamics, balances, negotiated orders, stories, and cultures.^15,44^ The results could be difficult to predict, may differ according to the particularity of the contexts and the environments, and can be surprising because of the dynamics characterizing complex adaptive systems.^15^ Negative UCs of telehealth have often been at the origin of, or associated with, difficulties in terms of acceptance, adoption, utilization, sustainability, and scaling-up of telehealth projects, whereas positive UCs have helped to increase the relevance and usefulness of some technological applications and their potential for adoption and utilization. The projects analyzed showed significant unintended systemic implications that a “technocentric” approach, which states that previously demonstrated or theoretically effective technology will be automatically used and have the expected impacts and benefits, could not anticipate. Interestingly, we notice that projects initiated almost 25 years ago raised issues and challenges that are still present in more recent projects. This observation could indicate that given the complexity and the perspective of such projects, their UCs continue to be underestimated.

Our study provides a comprehensive overview of the multidimensional UCs associated with telehealth. Indeed, beyond some UCs previously reported in the literature (eg, technology dependence, alert-fatigue, cognitive overload, stress and anxiety, frustration caused by technology rigidity, unadapted workflows, increased workload, appearance of new errors, etc),^6,11,15-20^ this study sheds light on four systemic dimensions that deserve more attention.

First of all, utilization of the technology, anticipated or otherwise, remains one of the most important issues. In all projects, telehealth utilization has necessitated many adjustments, changes, and negotiations that were rarely foreseen in the planning and initial phases of the projects. For example, in telepathology, the scientific evidence evolution and the emergence of new unplanned clinical utilization of technology (eg, cytology, teleautopsy, and emergency biopsies) had a great impact on existing dynamics and frameworks (eg, organizational, professional, legal, economic) and on the scope and the nature of the project.^8^

Second, telehealth has also resulted in the emergence of new service corridors that have disrupted existing ones much more than expected. These changes were often associated with new needs for organizations, particularly in terms of medico-administrative staff, investments, and resources to respond to new demands or activities. In addition, telehealth may have caused fragmentation of some services, especially when projects were planned on a specialty or subspecialty basis, without an overview of the organization of services and the interconnections between different specialties and levels of services involved (silos). A notable exception is the Magdalen Islands’ telehealth project, which was designed based on clinical needs from the whole organization and existing service corridors.^45^ In the same vein, telehealth may also challenge the delicate balance between the need for standardization of practices, corridors, and protocols and the need to keep some leeway for local creativity and innovation – challenging traditional top down bureaucratic approaches – in addition to respecting existing collaborative networks.^8,46,47^

Third, the issue of power relations was central in all projects: between different professional groups, between organizations, between professionals and organizations, between organizations and technology providers, and between different governance levels or sectors. For example, the prestige associated with the status of reference centre in a telehealth network and the image of “poor medicine” associated with service-seeking organizations illustrate the tension when telehealth introduces a new dynamic between organizations. Power issues were also present in the political sphere because telehealth triggered the delicate question of federal interference in policies that are under provincial jurisdiction. Issues related to power relations have greatly contributed to the difficulties or even failures of some telehealth projects.^8,13^

Fourth, telehealth involves the direct or indirect intervention of a multitude of actors, human and non-human alike. This situation could lead to significant changes in the responsibilities of each individual actor in the supply chain of services. The multiplication of standards, norms, rules, and laws that should regulate the practice of telehealth can challenge current frameworks that often are not well adapted to the new realities brought about by telehealth and e-health in general. For instance, the issue of services delivered by providers located in other jurisdictions or countries created challenges such as the financial flows of public health systems (eg, the reimbursement of outsourced telehealth services by public insurance to service providers located in other jurisdictions or countries), patient protection, quality of the medical act, insurance, and reimbursement.

Otherwise, it should be pointed out that among the ten projects studied, only 3 are still functional (A, C, D), 5 have ended (B, E, F, H, J), and 2 continue with very minimal or even episodic activity (G, I). The multidimensional UCs of telehealth have partly contributed to this result. This demonstrates the importance of a systematic and holistic approach to considering UCs, from the planning phase of telehealth projects until their scaling-up. In addition, it underscores the need to develop a comprehensive and critical vision of what telehealth can do, and also what it cannot, and a sense of which conditions determine these positive and negative outcomes.^20^ In other words, it is worth considering that telehealth does not exist, and cannot be defined, outside the environments where it is implemented and without taking into account its final recipients (individuals, groups, organizations, and communities).^48^

Similarly, our results report a number of issues encountered in other countries, even some that have a great tradition in telehealth.^5,9,10,49^ They also echo those of several authors who pointed out that the partial success or failure of telehealth initiatives are mainly due to the underestimation of the complexity of certain phenomena or changes, hence the importance of a holistic approach.^9,10,13,48^ In this regard, there is a growing agreement that there is a need to understand and consider the various dimensions and factors that influence the implementation, adoption, use, sustainability and scaling-up of telehealth, namely: socio-political, economic, regulatory, organizational, clinical, professional, human, legal, strategic, and governance aspects.^5,8,10,50-55^ Thus, our findings support the importance of applying best practices for telehealth implementation, including needs and e-readiness assessment, business case and budget, change management strategy, alignment with legal and regulatory policy, project evaluation, refinement and subsequent monitoring.^5,56-65^

Finally, as noted in this work, the introduction of telehealth into health organizations and systems remains a complex enterprise. Indeed, the health system is composed of a multitude of actors and levels of decisions with often poorly defined contours, interactions and interdependencies, which are very difficult to predict, manage or control.^49^ Telehealth UCs are one of the most obvious illustrations. Many of these UCs could not be avoided, but many others could be better understood or anticipated by the availability of more systematic strategic project planning and management.^59,66-68^ Indeed, it could be argued that some of the UCs identified in this work could be the result of bad or poor project planning (eg, the development of silos of information as a consequence of incompatible data management systems within the same service). This is partly true if we accept that some projects have been major experiments, and others emerged from funding opportunities or top-down political decisions, which led to not enough time or synergy between different governance levels or flexibility for local teams to better plan and consolidate the projects. However, because of the complexity of the changes and transformations observed at all levels, it would be ambitious to say that good planning alone could solve all the UCs. This is where an emerging and agile approach in projects (vs. the traditional approach where everything is planned and scheduled before the start of the project and leaves no room for unforeseen events) could be an excellent lever, not to anticipate all UCs, but to create a culture and dynamics that allow stakeholders to address some negative UCs, or temper their impacts, but also to capitalize on the positive UCs that could occur as well. In fact, rigid project planning could have the opposite effect, because not every UC is a problem of planning, but requires cultures and dynamics to cope with the unforeseen in increasingly complex telehealth projects. Therefore, organizational and systemic flexibility is also needed to allow field teams to manage these UCs.^8,13,49^

### Future Research and Evaluations: The Need for a Holistic Approach to Telehealth

Our study highlights the need and relevance of having a detailed understanding of the human, professional, technological, organizational, economic, legal, ethical, socio-political, cultural, and societal implications of telehealth. Thus, telehealth evaluation should go beyond the mere processes of technical reasoning which, albeit important, are often limited to the evaluation of expected effects and benefits. Such a holistic approach would help to better account for the contexts and environments where telehealth is implemented and used, along with their evolution over time.^8-10,13^ This perspective would also help to mitigate and correct some of the UCs, or develop strategies to deal with them during the project, because they cannot be completely avoided in complex health organizations and systems.^11^

Future research-evaluation studies should thus be more sensitive to the complexity associated with the use of telehealth. This requires continuous and repeated evaluations (reevaluation or reassessment) of how telehealth is used and perceived, as well as its evolution in various environments and over time. In this regard, the dynamic and holistic framework developed by Greenhalgh et al is a very promising attempt to account for such complexity.^9^ In addition, the use of a conceptual UCs framework, such as proposed by Bloomrosen et al as a complementary or integrated tool for these evaluations, would make it possible to better understand the multidimensional and interdependent factors that influence implementation and utilization of telehealth, along with its impacts, intended or otherwise, at all levels.^11^

### Strengths and Limitations

Given the importance of the context in which telehealth projects take place, the main limitation of this study concerns the generalization of its findings to other countries. Therefore, a comparison of UCs related to Quebec telehealth projects with those in other countries would be relevant and useful. Another limitation is the fact that we did not try to count the frequency or put a weight on the importance of one UCs domain vs. another. Due to its qualitative nature, this study aimed to provide a rich and detailed analytical description and to offer a first systemic and multidimensional portrait of telehealth UCs that goes beyond the sole technological dimension.^20,69,70^ In addition, our systemic and longitudinal approach (over a 22-year period) helped to increase the reliability of results. The fact that the projects analyzed were initiated in the same institutional context increases the consistency and reliability of our observations; this characteristic makes it possible to remedy the problem of comparing programs and projects from different contexts.^31^

Moreover, the fact that this study was based on secondary analysis of data originally collected for other purposes is a limitation. The unavailability of certain data on the steps of design and planning of projects could be a weakness of this work. However, the authors participated in the various evaluations, which makes it possible to overcome, at least partially, the limits that secondary data may present. Using secondary analysis is above all pragmatic since it allows exploiting data that are difficult to collect in a primary way. It could constitute an exploratory step prior to further studies on this issue.^29,30^ Thus, even though it provides no “recipe for generalization,” this study enables more productive debate about telehealth projects and initiatives.^10^

This work also presents other limitations. Its retrospective nature meant that we were not completely able to make a clear distinction between the “unintended” and “unanticipated” consequences. According to Bloomrosen and al, “*The ‘unintended’ implies lack of purposeful action or causation, while the ‘unanticipated’ means an inability to forecast what eventually occurred.*” They acknowledge that there are definitional and typological issues that need to be addressed. Some of what have been called “unintended consequences” would rather be classified as “unanticipated consequences.” This said, this paper also raises the need for more methodological, conceptual and theoretical work on UCs of health-ITs (and innovations in general), and particularly to propose a clear typology of the different types of consequences of the implementation of health-ITs. Our study could thus be a practical basis for future works on the topic.

## Conclusion

By uncovering some of the UCs of telehealth, this study emphasizes the importance of addressing the development, implementation, and use of telehealth from a holistic and multilevel perspective. The dominance of a tradition of technology-centric evaluation, which underestimates the contexts and complexity of processes, interactions, and interdependencies characterizing health organizations and systems, partly explains the current situation where telehealth is far from fulfilling expectations built on its potential. This picture is reflected in the striking contrast between the “theoretical” telehealth utility and contribution and the “disappointing” reality or perception of its use and integration into several health systems today.

Telehealth offers significant potential for improving access, quality, continuity, and integration of health services for the benefit of the population. At the same time, it raises questions and issues relating to usages, transformations, symbols, or representations not initially suspected. Thus, an inclusive and collaborative approach that engages all relevant stakeholders, including patients-citizens and communities, should be adopted in the design, planning, implementation, and evaluation of telehealth services. Finally, it is important to learn from these projects, both successes and failures, in a process of sharing experiences to inform decision-making and help to translate knowledge into action.

## Acknowledgments

The authors would like to thank all people, organizations and institutions that have contributed to the evaluations used in this study.

HA was supported by doctoral scholarships from: (1) the Strategic Training Fellow in Transdisciplinary Research on Public Health Interventions “Promotion, Prevention and Public Policy (4P)” of the Canadian Institutes of Health Research, Ottawa, ON, Canada and the Quebec Population Health Research Network, Montréal, QC, Canada; and (2) the Research Center on Healthcare and Services in Primary Care of Laval University, Quebec City, QC, Canada.

## Ethical issues

This study did not require the participation of human subjects. The information was anonymous and no data related to individuals were collected or are accessible. As a secondary analysis of existing data, ethics approval was therefore not necessary.

## Competing interests

Authors declare that they have no competing interests.

## Authors’ contributions

HA, MPG, and JPF conceived and designed the study and were involved in data collection, analysis, and interpretation of results. HA produced the first draft of this manuscript, and received input from MPG and JPF. All authors read and approved the final manuscript.

## Authors’ affiliations

^1^Institute of Health and Social Services in Primary Care, Research Center on Healthcare and Services in Primary Care, Laval University, Quebec City, QC, Canada. ^2^Research Center of Quebec City University Hospital Center, St-François d’Assise Hospital, Quebec City, QC, Canada. ^3^Faculty of Nursing Science, Laval University, Quebec City, QC, Canada. ^4^Department of Social and Preventive Medicine, Faculty of Medicine, Laval University, Quebec City, QC, Canada.

## Key messages

**Implications for policy makers**

- Telehealth is primarily a health system transformation challenge, not just a “technological device” implementation process. Its use is associated with systemic and multidimensional impacts and changes at several levels: sociopolitical, economical, organizational, professional, cultural, human, legal, technological, and governance.
- Decision-makers should be more sensitive to the complexity associated with telehealth in order to account for the multidimensional and interdependent factors that influence its implementation and utilization, as well as its impacts, intended or unintended, at all levels.
- The predominance of a “technocentric” vision, which claims that a demonstrated effective technology in one context will be automatically used and have the same effectiveness elsewhere, could explain the contrast between the stated benefits of telehealth and its low degree of integration in many contexts.

**Implications for public**

The unintended consequences (UCs) of telehealth, which may be critical for patients or communities in some cases, should better be taken into account by decision-makers so that it can really contribute to improving access, continuity and quality of healthcare and services for citizens.
